# Intrusion Detection Framework for Internet of Things with Rule Induction for Model Explanation

**DOI:** 10.3390/s25061845

**Published:** 2025-03-16

**Authors:** Kayode S. Adewole, Andreas Jacobsson, Paul Davidsson

**Affiliations:** 1 Department of Computer Science and Media Technology, Malmö University, 205 06 Malmö, Sweden; andreas.jacobsson@mau.se (A.J.); paul.davidsson@mau.se (P.D.); 2 Sustainable Digitalisation Research Centre, Malmö University, 205 06 Malmö, Sweden

**Keywords:** security, intrusion detection, IDS, explainability, ensemble models, feature selection, machine learning, rule induction

## Abstract

As the proliferation of Internet of Things (IoT) devices grows, challenges in security, privacy, and interoperability become increasingly significant. IoT devices often have resource constraints, such as limited computational power, energy efficiency, bandwidth, and storage, making it difficult to implement advanced security measures. Additionally, the diversity of IoT devices creates vulnerabilities and threats that attackers can exploit, including spoofing, routing, man-in-the-middle, and denial-of-service. To address these evolving threats, Intrusion Detection Systems (IDSs) have become a vital solution. IDS actively monitors network traffic, analyzing incoming and outgoing data to detect potential security breaches, ensuring IoT systems remain safeguarded against malicious activity. This study introduces an IDS framework that integrates ensemble learning with rule induction for enhanced model explainability. We study the performance of five ensemble algorithms (Random Forest, AdaBoost, XGBoost, LightGBM, and CatBoost) for developing effective IDS for IoT. The results show that XGBoost outperformed the other ensemble algorithms on two publicly available datasets for intrusion detection. XGBoost achieved 99.91% accuracy and 99.88% AUC-ROC on the CIC-IDS2017 dataset, as well as 98.54% accuracy and 93.06% AUC-ROC on the CICIoT2023 dataset, respectively. We integrate model explainability to provide transparent IDS system using a rule induction method. The experimental results confirm the efficacy of the proposed approach for providing a lightweight, transparent, and trustworthy IDS system that supports security analysts, end-users, and different stakeholders when making decisions regarding intrusion and non-intrusion events.

## 1. Introduction

Our world today is surrounded by a vast array of electronic devices that are revolutionizing how we live and work. In this context, the IoT is emerging as a groundbreaking technology that is reshaping industries and making life smarter through intelligent, and highly connected devices. IoT is enhancing areas like healthcare monitoring, environmental tracking, water management, smart agriculture, and smart homes [1,2]. Specifically, IoT enables diverse physical devices to communicate and collaborate with one another, seamlessly transferring data across numerous networks without the need for human intervention [3,4]. IoT is embedded with several components such as sensors, actuators, software, processors, and many other technologies which enable devices and systems to connect and communicate over the Internet. According to [5], it is estimated that by the year 2030, the number of connected IoT devices will reach 29 billion, signifying a difference of about 20.4 billion compared to 2019. This surge is attributed to the growing dependence of households and businesses on embedded technologies. In terms of sector-specific distribution, the industrial and manufacturing domain leads in IoT device adoption, accounting for 40.2% of the total. The medical sector closely follows, constituting 30.3% of IoT devices usage. Other sectors include retail, contributing 8.3%, security with 7.7%, and transportation with 4.1% of the overall distribution of IoT equipment [6]. This surge is further fueled by the widespread adoption of Internet connectivity and the emergence of innovative technologies such as cloud, edge, and fog computing.

The increasing inter-connectivity of these devices also brings forth unprecedented security challenges, particularly in safeguarding IoT networks from malicious activities. One of the paramount concerns is the timely and accurate detection of intrusion within IoT networks, a pivotal aspect in ensuring the integrity and security of these interconnected systems [7,8]. Interconnected IoT devices have become susceptible targets for malicious threats due to their operation with low power and computation management. Distributed Denial of Service (DDoS) and eavesdropping are some of the most catastrophic attacks against IoT [9]. Traditional security methods, such as encryption, firewalls, and access control, often prove ineffective in securing these devices; they become time-consuming and insufficient in vast networks with numerous interconnected devices where each component introduces its own vulnerability, challenging the effectiveness of traditional security measures [3]. An additional layer of defense for safeguarding an IoT network against security attacks can be achieved through the implementation of an Intrusion Detection System (IDS), serving as a secondary line of defense [10].

An IDS is a highly effective tool for monitoring and analyzing a device’s behavior. IDS is commonly used as an additional layer of security to safeguard systems and networks. They monitor events for suspicious activity, offering early warnings of potential threats. This proactive approach helps reduce damage and limit the impact of successful intrusion attempts. As an extra line of defense, IDSs play a crucial role in maintaining the security and integrity of networks and systems [11]. By mapping user-behavior patterns on the network, IDS can identify abnormal activities, indicating a deviation from normal device or user behavior. IDS can continuously scan events across various network systems, analyzing them to detect behavioral changes and uncover potential security incidents with the aim of providing timely responses to threats [10]. Intrusion detection is determined by its deployment method and detection methodology. Depending on deployment, an IDS can be either host-based, network-based, or hybrid-based. In terms of detection method, it may be signature-based, anomaly detection-based, statistical-based, rule-based, state-based, heuristic-based, specification-based, or hybrid detection [3,12]. In response to the challenges of detecting intrusion, machine learning (ML) emerges as a promising method, capable of discerning irregular patterns in network behaviour. There are many solutions in the literature that utilise ML methods to detect IoT network intrusion [13,14,15]. In this domain, ensemble-based methods have been identified as promising approaches for developing robust IDS (see, for example, the studies in [3,7,10,13,16,17,18]). For instance, the authors of [3] developed an ensemble model for IDS that leverages ML methods such as Logistic Regression (LR), Naive Bayes (NB), and decision tree (DT). Using the CICIDS2017 intrusion detection dataset, the ensemble model achieved an accuracy of 88.96% and 88.92% for multi-class and binary classification respectively. Awotunde et al. [7] propose ensemble methods that considered XGBoost, bagging, Extra Trees, Random Forest, and AdaBoost using ToN-IoT datasets. Seven telemetry datasets, including Fridge, Thermostat, GPS Tracker, Modbus, Motion Light, Garage Door, and Weather are explored to develop the ensemble models. Hazman et al. [19] proposed an IDS framework that employed Boruta-based feature-selection method. The features selected from Boruta were used to train an AdaBoost ensemble algorithm based on two IDS datasets (i.e., NSL-KDD and BoT-IoT). This approach is based on a binary classification problem and does not consider multi-class classification tasks. Even though the model produces promising results, Boruta feature selection was time consuming, which may not be applicable for real-time IoT settings. In [18], an ensemble model that leverages DT, Rprop MLP, and LR was developed using voting and stacking methods. Feature-selection methods using correlation-coefficient scoring and recursive feature elimination (RFE) were employed to select important features for IDS. Synthetic Minority Oversampling Technique (SMOTE) was utilized to balance the distribution of samples per class in the BoT-IoT and TON-IoT datasets used in the study. This approach considers only the binary classification task and neglect multi-class classification.

Although many ensemble-based methods have been proposed for IDS development, these studies neglect the fact that the transparency of IDS models plays a significant role in IDS adoption by stakeholders. Explainable IDS will assist security analysts to understand what constitutes abnormal behaviour in IoT network and what needs to be done to proactively mitigate future attacks. In some use cases, such as law enforcement, criminal investigation, and forensic analysis, where IDS is considered as a useful tool to understand digital evidence, explainable IDS is worth considering. Gaining insights into the reason and logic behind the decision of an IDS to flag a specific event as intrusion can aid security practitioners in assessing the system’s effectiveness and creating more cyber-resilient solutions. While some studies considered multi-class classification problems, others neglect the need to explore such approach for developing effective IDS solutions. Considering the resource-constrained nature of IoT devices, the need for real-time detection capability and a lightweight security framework has become necessary. This paper aims to address the aforementioned research issues identified in the previous studies. In this paper, we propose a framework that integrates transparent ensemble methods that do not only detect intrusion based on binary and multi-class classifications, but also leverage a rule-induction approach to provide explainability. More clearly, this paper contributes in the following ways:

- It investigates the performance of five ensemble methods to develop an efficient IDS solution for IoT network environment;
- It integrates rule induction for knowledge extraction to provide explanations for the outcomes of the ensemble models, thus aiding better understanding of the models’ predictions;
- It provides extensive evaluation results based on two network-intrusion datasets.

The remaining parts of this paper are organized as follows. Section 2 focuses on related works in intrusion detection for IoT. Section 3 provides a detailed discussion on the methodology used in our study, including a discussion of the ensemble methods, and the rule-induction method for model explanations. Section 4 presents a comprehensive discussion of the various results obtained, how we compare the proposed methods with the related studies in the domain of IDS development for IoT, and the limitations of our study. Finally, Section 5 concludes the paper and offers future research directions.

## 2. Related Works

This section provides a comprehensive review of existing studies that focused on IDS development for IoT networks. As observed, several studies focused on ML methods such as traditional, ensemble, and deep learning approaches.

Yakub et al. [20] proposed a ML-based IDS for IoT network attacks, employing the UNSW-NB15 dataset. The research focused on feature scaling using Min–Max normalization and PCA for feature selection. Six ML models, including XGBoost and CatBoost, were evaluated, with PCA-XGBoost exhibiting the highest accuracy of 99.99%. The study emphasizes the reduction of communication overhead, and the proposed system proves effective in detecting various network-attack scenarios. The authors suggest adopting an ensemble model with a novel dataset suitable for the IoT environment to design IDS that is tailored to the IoT situation. The study in [9] investigates ML classification algorithms to secure IoT against DoS attacks, emphasizing anomaly-based IDS. Various classifiers, such as RF and Extreme Gradient Boosting, were tested on the CIDDS-001, UNSW-NB15, and NSL-KDD datasets. The paper introduces statistical assessments using Friedman and Nemenyi tests, shedding light on the performance of classifiers. The performance results and statistical tests reveal that classification and regression trees and the Extreme Gradient Boosting classifier show the best trade-off between prominent metrics and response time, making them suitable choice for building IoT-specific anomaly-based IDSs.

Diro et al. [21] conducted an extensive literature review of anomaly detection in IoT. They highlight challenges in securing heterogeneous IoT devices and propose the integration of blockchain for collaborative learning. The paper evaluates supervised ML algorithms like K-Nearest Neighbour (KNN) and unsupervised approaches, emphasizing the drawbacks of classical algorithms and resource limitations in IoT. Banaamah et al. [22] focused on deep learning models, including Convolutional Neural Network (CNN), Long Short-Term Memory (LSTM), and Gated Recurrent Unit (GRU), for IoT intrusion detection. While highlighting the benefits of deep learning, the study acknowledges challenges, such as the need for massive training data. The proposed methods demonstrate high accuracy, with LSTM at 99.8% and a false alarm of 0.02%, outperforming CNN with 99.7% and a false alarm of 0.03, while GRU achieved 99.6% with a false alarm of 0.04%. However, the study notes limitations related to network load and execution-time increase with a Deep Neural Network (DNN). Saba et al. [23] addressed the security challenges posed by IoT and proposed a CNN-based approach for anomaly-based IDS tailored for IoT environments. The study emphasizes the inadequacy of traditional intrusion detection technologies due to resource constraints in IoT devices. Their research contributes by categorizing attacks through deep learning, proposing an IDS framework for IoT networks. The CNN model, validated on the BoT-IoT dataset, demonstrates accuracy of 95.55% in classifying various traffic types. However, the authors acknowledge that significant research is still required for advancements in IoT to have a better threat-detection rate with IoT progression in the industry, suggesting the potential for refining the model.

The DNN solution has also been studied by Ahmad et al. [12]. The approach explores various deep learning models based on the IoT-Botnet 2020 dataset. The study underscores the efficiency of DNN in learning complex features and achieving a high detection accuracy of 99.01% with a false-alarm rate of 3.9%. Notably, the authors identify a challenge in detecting minority-class labels efficiently, highlighting a potential area for future research in multi-class classification scenarios. Sarhan et al. [24] explored the impact of feature dimensions on classification performance across diverse datasets. Their study addresses the unreliability of Network Intrusion Detection Systems (NIDS) in IoT networks, focusing on the generalizability of feature-extraction algorithms and ML models across different NIDS datasets. The authors advocate for further investigation into finding an optimal feature-selection approach and ML classifiers. Susilo et al. [25] proposed a DNN method as a means to enhance IoT security, focusing on the detection of DoS attacks. The study employed BoT-IoT dataset by evaluating RF, CNN, and Multi-Layer Perceptron (MLP) algorithms. RF and CNN provided the best results in terms of AUC-ROC metric. The authors discuss the potential of integrating these algorithms into NIDS for real-time mitigation. Hanif et al. [26] addressed authentication challenges in IoT by proposing an Artificial Neural Network (ANN)-based threat-detection model. The proposed ANN model, trained on the UNSW-NB15 dataset, achieved 84% accuracy and an average False-Positive Rate (FPR) of 8% in classifying diverse attacks. The challenges in this study relate to low accuracy and high false alarm.

In [27], a three-layer IDS for IoT devices in smart homes is proposed, employing a supervised ML approach. The IDS distinguishes between benign and malicious network activities, identifies malicious packets, and classifies the type of attack. The study evaluates the system on a smart-home test bed with popular IoT devices, showcasing its ability to automatically detect and classify attacks. A DT based on the J48 algorithm emerges as a promising classifier. J48 achieved an F-measure of 99.7%, 97.0%, and 99.0% across all three experiments.

Abbas et al. [3] proposed an ensemble model for IDS that considers LR, NB, and DT algorithms. The ensemble model achieved 88.96% and 88.92% accuracy for multi-class and binary classification, respectively, based on CICIDS2017 dataset. The performance of different ensemble methods using bagging and boosting DT techniques was explored in [10]. The authors observed that Light Gradient Boosting (LGB) algorithm produced the best results in terms of speed and AUC-ROC score for multi-class IDS solution. Awotunde et al. [7] developed ensemble models based on XGBoost, bagging, Extra Trees, Random Forest, and AdaBoost using thw ToN-IoT dataset. Odeh et al. [17] proposed an ensemble model that utilize voting policy to integrate deep learning models such as CNNs, LSTM, and GRUs.

Danso et al. [13] utilized a SelectKbest feature-selection approach combined with Chi-Squared to identify discriminating features for IDS. ML algorithms such as kNN, Support Vector Classification (SVC), and NB were combined to develop an ensemble model using voting, stacking, boosting methods. It was observed that the accuracy of the stacking method (i.e., 99.87%) on CICIDS2017 gave the best performance, but performs the least well in precision and FPR when compared with the bagging method for the classification task investigated in the study. In [16], an ensemble model for binary classification of IDS is proposed. The approach utilize voting and stacking methods for RF, DT, LR, and kNN algorithms, achieving accuracy of 98.63% based on the stacking method.

Hazman et al. [19] proposed an IDS framework that employed a Boruta-based feature-selection method. The features selected from Boruta were used to train an AdaBoost ensemble algorithm based on NSL-KDD and BoT-IoT IDS datasets. This approach is based on a binary classification problem and does not consider a multi-class classification task. One limitation of this approach is the computational complexity of the Boruta feature-selection approach for IoT intrusion detection in real-time environment. In [18], an ensemble model based on DT, Rprop MLP, and LR was developed using voting and stacking methods. Feature-selection methods using correlation-coefficient scoring and RFE were employed to select important features for IDS. To address a class imbalance problem, the study utilized the SMOTE technique on both BoT-IoT and TON-IoT datasets. The study considers only the binary classification task and neglects multi-class classification, which is also beneficial for developing effective IDS.

Hassan et al. [28] incorporated Local Interpretable Model-agnostic Explanations (LIME) and SHapley Additive exPlanations (SHAP) to provide model explanations for IDS in a Vehicular Ad-hoc Networks (VANETs) environment. The study leveraged a machine learning pipeline that consider correlation-based feature selection and a Random Forest classifier to achieve a classification accuracy of 100% for identifying normal and malicious traffic in VANETs. An explanation of the rules for security threats, based on a natural language-generation strategy using hybrid prompt learning, has been given in [29]. The goal of the study was to prevent the privacy and security risks associated with automation rules that may potentially compromise the security of a trigger-action systems such as IoT.

In contrast to the existing studies, this paper proposes an IDS framework that integrates model explainability for transparency and accountability, which is achieved through the integration of a rule-induction method. We investigate five lightweight ensemble methods to address the resource-constrained nature of IoT devices. This will subsequently support the development of real-time IDS solutions. The proposed approach can help security analysts and end-users to understand what constitutes abnormal behaviors in IoT networks, as well as how to deploy mitigation strategies to reduce threat impacts. In this study, we consider both binary and multi-class classification problems and provide an explanation for each category of classification task.

## 3. Materials and Methods

**Figure 1:**
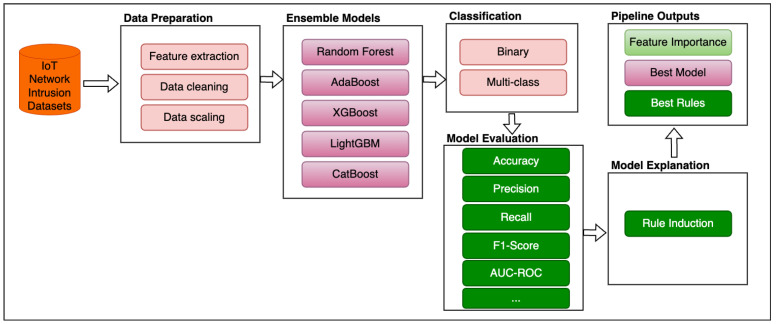
Proposed framework for intrusion detection in IoT networks.

**Figure 2:**
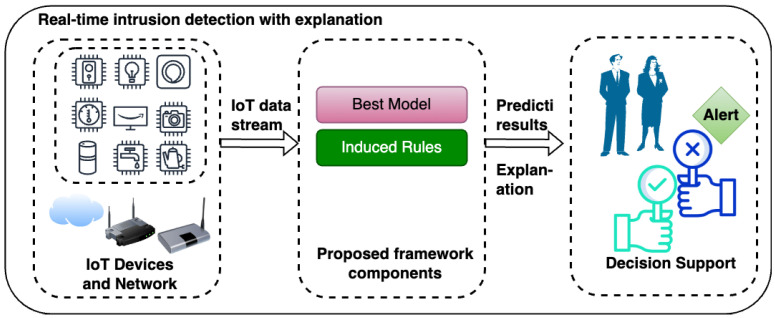
Real-time IDS with explainability.

This section provides a detailed discussion on the different components of the proposed framework for IDS in this study. Different methods have been investigated to achieve the objectives of this research, as highlighted in Section 1. Figure 1 shows the main pipeline of the proposed framework. It consists of data collection from the IoT network environment, data preparation, ensemble models, classification type, model evaluation, and model explanation. Each stage is described in detail in the subsequent sections. The main focus of the proposed framework is to develop IDSs that leverage the feature importance of decision-tree-based models integrated in the ensemble algorithms to provide model explanations and transparency. The proposed framework also targets both binary and multi-class classification based on ensemble learning methods. As shown in Figure 2, the best model according to the proposed framework is then used to detect intrusion in IoT environment. In addition to detecting normal and attack cases using the best proposed ensemble model, the rule induced from the real-time intrusion detection can be used to further aid decision making by IoT security analysts and end-users.

### 3.1. Dataset Description

This study utilized two publicly available datasets that have been widely used in the literature for intrusion detection in IoT networks. The first dataset is CIC-IDS2017 [30], which can be downloaded at https://www.unb.ca/cic/datasets/ids-2017.html (accessed on 24 December 2024). This dataset contains 2,830,743 samples with 79 features, including the label features that described both normal samples and the attack cases. There are 15 class labels, including the normal case. The attack scenarios range from DDoS, DoS Hulk, DoS GoldenEye, DoS slowloris, web-attack brute force, and so on, as presented in Table 1. This table shows the class distribution in the CIC-IDS2017 dataset with the benign (normal) class having the largest proportion, and this is followed by the DOS Hulk attack.

The second dataset is the CICIoT2023 that is publicly available for download at https://www.unb.ca/cic/datasets/iotdataset-2023.html (accessed on 24 December 2024). CICIoT2023 is a large-scale dataset for testing a wide range of network intrusion attacks. This dataset contains 45,019,234 samples with 40 features, including the label features that describe both normal (i.e., BENIGN) samples and attack cases (see Table 2). There are 33 attack cases in the dataset, with DDoS-ICMP-Flood constituting the largest proportion (15.312%), as shown in Table 2. Uploading attack has the lowest proportion according to the table. For a detailed discussion of the different attacks in the dataset, the reader is referred to [31].

There are 14 and 33 attack cases in CIC-IDS2017 and CICIoT2023, respectively. According to Table 1 and Table 2, some of these attack are grouped as follows:

- Backdoor malware: This is a typical web-based attack that enables the attacker to use backdoor malware to gain unauthorized access to IoT devices. The malware, usually called a “backdoor”, hides itself such that the attacker can use an undisclosed entry point to gain access to the system. A typical example is the backdoor malware in the CICIoT2023 dataset.
- Botnet: Botnet attacks involve the use of a command and control architecture, which permits the attacker to take control of operations from vulnerable IoT devices called “zombie bots” from a remote location. This attack can be used to launch DDoS and other exploitations.
- Browser hijacking: This is a web-based application attack that allows attacker to modify the browser settings. This includes changing the default home page, setting a default search engine, and bookmarking certain websites to take advantage of unwanted advertisements [31].
- Command Injection: This attack is an attack against web/mobile applications, such as the one used to control IoT devices that injects malicious commands into the application input field. The objective is to have unauthorized access to sensitive parts of the application.
- Denial of Service (DoS)/Distributed Denial of Service (DDoS): While a DoS attack uses a single machine to compromise the target device, DDoS, on the other hand, leverages multiple machines in a coordinated manner to execute a denial of the service attack targeting the victim’s device. For instance, in [31], one Raspberry Pi machine executed a flooding attack against IoT devices, while multiple Raspberry Pis were employed to execute DDoS attacks using an SSH-based master–client setup. This type of attack has different variations, including DDoS-ICMP-Flood, DDoS-UDP-flood, DDoS-TCP-flood, DDoS-SYN-flood, among others (see Table 1 and Table 2).
- Brute force: This attack uses a combination of characters in several trials to crack passwords or login credentials. When a wordlist is used to aid in slowing down the time that would be taken by a brute force, then such an attack is called a dictionary attack. One of the vulnerabilities of IoT devices is a weak password [32], which is leveraged by attacker to execute a brute-force attack against the target login platform.
- Domain Name Server (DNS) Spoofing: DNS spoofing attack, also called a DNS cache-poisoning attack, is used by the attacker to manipulate DNS records to redirect legitimate users or requests to malicious websites that mimic the users’ intended destination. The attack enables the attacker to gain access to sensitive information and spread malicious software.
- Mirai Botnet: This is a DDoS attack that targets thousands of IoT devices such as printers, baby monitors, IP cameras, and so on, turning them into malicious botnets or Zombie bots [33].
- PortScan: This attack is used by the attacker to determine which port is open, closed, or filtered. It is a type of information-gathering attack or reconnaissance attack in which a series of packets is sent to a target machine to ascertain the status of the ports running on that machine. A tool such as Nmap [34] can be used for this purpose. In the CICIoT2023 dataset, this attack is referred to as vulnerability scan [31].
- Reconnaissance: A reconnaissance attack is an information-gathering attack which allows the attacker to gather data before the actual attack is executed. This can help the attacker gain more insights on the target machine as to what needs to be done to execute successful attack.
- SQL Injection: In SQL injection, an attacker uses an SQL query in the input fields of the web application. This causes the application to disclose sensitive information from the database. This attack can be used to execute commands on the database server.
- Uploading: Attackers use uploading attack to leverage vulnerabilities related to web application, which allows for the uploading of malicious files such as malware code, backdoor, and so on to gain unauthorized access on the target application.
- Cross-site Scripting (XSS): This attack enables the attacker to inject malicious client-side script into a web application that control the IoT devices. This script is executed by the user’s browser and it allows the attacker to steal sensitive information such as cookies, session tokens, and other sensitive data.

### 3.2. Data Preparation

Data preparation is a crucial step in developing ML models for IDS. In this study, this stage involves extracting relevant features from the dataset, data cleaning, and data scaling. In the first case, we used the features extracted from the dataset as provided by the authors of the datasets. This phase involves extracting relevant features from the raw packet data that are collected from the IoT network environment. In the case of CICIoT2023, 33 attacks were implemented in an IoT topology comprising 105 devices. Basically, the raw data are in the form of **.pcap** files processed to extract relevant features for data analysis [31]. After feature extraction, we performed data cleaning.

Data cleaning is an important step to ensure that the data used to develop machine learning models for IDS is free from redundant information in order to expedite the learning process when employing ML algorithms. This process basically involves removing irrelevant features that could negatively impact model performance, converting categorical features to numerical features, and addressing the missing values. In the case of CIC-IDS2017, one duplicate feature (i.e., Fwd Header Length) was removed from the dataset, leaving 77 features excluding the class label. Two features, FlowBytes and FlowPackets, have infinity values and these were converted to NaN. Furthermore, there are 308,381 duplicate samples out of 2,830,743 total samples in the dataset after merging the different files in the MachineLearningCVE directory. These duplicate values were removed and all missing values were filled using kNN imputation with 2 neighbours. The proportion of the missing values constitute 3128 samples after dropping the duplicate rows. Label encoding was employed to encode the class label (i.e., the target variable). The label-encoding method involves converting categorical variables into discrete numerical values. For instance, the benign class is assigned a numeric value of 0 while an attack is given a class of 1, as in the case of binary classification.

For the CICIoT2023 dataset, the data cleaning involves removing a total of 24,013,505 duplicate samples from the the dataset. This is to avoid bias and over-fitting during training and to ensure that the trained model can generalize to unseen samples [35,36]. This dataset contains about 53.34% of duplicate samples. The rate feature has infinity values, which was replaced with NaN. Thereafter, all missing values were filled using using kNN imputation with two neighbours. The proportion of the missing values constitute 1526 samples. Similarly, we applied the label-encoding method to encode the class label. For the purpose of clarity, we focused only on the binary classification task for this dataset by assigning all intrusion samples into attack class. Thus, we have both benign and attack classes.

Furthermore, for data scaling, we used Min–Max data normalization technique to minimize model bias. This is necessary because some features may have significantly larger values than others, potentially leading to biased model results. Equation (1) shows how to compute the normalize values for the data using Min–Max which will generate new values for each column in the dataset in the close interval of [0, 1]. This technique ensures that features with smaller values are not overshadowed by those features that contain larger values for their columns, thereby producing a more balanced dataset for model development. For each dataset, a ratio of 70/30 percent is used for training and testing sets, respectively.

(1) $$x^{\prime }=\frac{x-\min(x)}{\max(x)-\min(x)}$$

where *x* is a specific value of a feature before scaling, min(x) and max(x) are the minimum and maximum observed value for the features, respectively, and $x^{\prime }$ is the new normalized value for the feature.

### 3.3. Ensemble Algorithms

**Figure 3:**
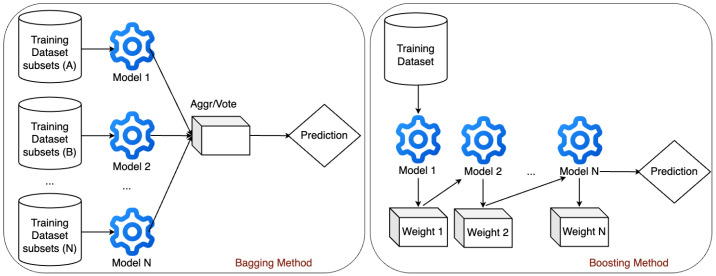
Selected ensemble methods.

As discussed earlier, this study adopted five ensemble algorithms to develop classification models for IDSs in an IoT environment. Ensemble algorithms are powerful ML algorithms that combine predictions from multiple models to improve overall performance and robustness [17]. By aggregating diverse models, ensemble algorithms reduce over-fitting, enhance accuracy, and handle data complexities better than individual models [16]. There are three main types, bagging, boosting, and stacking. Bagging (Bootstrap Aggregating) creates multiple models in parallel using random subsets of the data and aggregates their predictions. A typical example is Random Forest, which is a popular bagging algorithm. Boosting builds models sequentially, where each new model corrects the errors of the previous weak learners. Boosting algorithms include Gradient Boosting, AdaBoost, and XGBoost among others. They are widely used for reducing bias and variance, yielding highly accurate models. Both bagging and boosting employ homogeneous classifier to build the final predictive model; hence, they are termed homogeneous ensemble methods. Conversely, stacking ensemble algorithms combine predictions from multiple base models with different architecture (i.e., heterogeneous) using a meta-model learner. In this study, we employed five ensemble algorithms which belong to both bagging and boosting methods. Figure 3 gives the summary of the selected ensemble methods.

The five ensemble algorithms are Random Forest (bagging), AdaBoost (boosting), XGBoost (boosting), LightGBM (boosting), and CatBoost (boosting). The justification for adopting these classification methods is their performances as reported in different domains including IDS [16]. In addition, this study integrates model explainability in the proposed framework by leveraging ensemble methods that use decision trees as the default weak learners, where in this case bagging and boosting methods are ideal candidates for our selection.

#### 3.3.1. Random Forest

Random Forest is a highly effective and versatile machine learning algorithm known for its accuracy and adaptability to diverse datasets. As an ensemble method, it combines predictions from multiple decision trees using the bagging technique, where trees are trained on bootstrap subsets of data [10]. Each node in a tree selects the best predictor randomly, adding a layer of randomness that enhances robustness against over-fitting. Random Forest builds numerous decision trees, each contributing to the final prediction through majority voting for classification tasks. As the number of trees increases, the generalization error stabilizes, ensuring consistent performance. Notably, Random Forest often delivers promising results with minimal parameter tuning, such as controlling tree depth or the number of instances per node. Its resilience to noise and over-fitting makes it widely applicable across domains. For model training, Random Forest operates in O(t∗m∗n∗log(n)), where *t* is the number of trees in the forest, *m* is the number of features, and *n* is the number of training samples. This algorithm operates in O(t∗d) when used for inferencing (i.e., for classification), where *d* is the depth of the trees. This can be approximated to O(log(n)) in balanced trees. This complexity makes Random Forests efficient for many practical applications. Nevertheless, as the number of trees or the size of the dataset increases, the computational cost may become significant. Therefore, it is important to investigate both the training and inference times of each ensemble algorithm based on the two datasets employed in this study to understand their practical implications for IoT environments. In Section 4, we provide a detailed analysis of these training and inference times for each ensemble algorithm.

#### 3.3.2. AdaBoost

Adaptive Boosting (AdaBoost) is a an ensemble learning algorithm designed to improve weak learners by sequentially combining them into a strong classifier. Unlike Random Forest, AdaBoost assigns weights to training instances, focusing on samples misclassified by previous iterations as presented in Figure 3. Each weak learner is trained to correct these errors, and the final model aggregates predictions with weighted voting based on the learners’ accuracy [37]. AdaBoost dynamically adjusts to challenging data points, focusing on those harder to classify. While sensitive to noisy data and outliers, its adaptability and simplicity make it effective for binary and multi-class classification tasks. Adaboost has O(t∗m∗n) computational complexity for model training, where *t*, in this case, represents the number of weak learners, *n* is the number of training samples, and *m* is the number of features. For inference, the algorithm has O(t∗d) complexity, where *d* is the depth of the trees.

#### 3.3.3. XGBoost

Extreme Gradient Boosting (XGBoost) is a highly efficient and scalable ensemble algorithm designed for supervised learning tasks. It extends traditional boosting by employing a gradient-boosted decision tree (GBDT) framework optimized for performance and flexibility [38]. XGBoost builds models iteratively, where each new tree minimizes a custom loss function by adjusting weights and improving predictions. The objective function in XGBoost is a combination of loss and regularization as given in Equation (2)

(2) $$Obj=\sum_{i=1}^{n}L\left(y_{i},{\hat{y}}_{i}\right)+\sum_{i=k}^{K}\Omega \left(f_{k}\right)\;$$

where $L(y_{i},{\hat{y}}_{i}$ is the loss function, measuring the difference between predictions ${\hat{y}}_{i})$ and the actual targets $y_{i}$. $\Omega (f_{k})$ regularizes the complexity of the model as defined by:

(3) $$\Omega \left(f_{k}\right)=\gamma T+\frac{1}{2}\lambda {∥w∥}^{2}\;$$

where *T* is the number of leaves, *w* is the leaf weights, and γ and λ are regularization parameters. XGBoost uses techniques like tree pruning and parallel processing, and it can handle sparse data. XBGoost operates in O(t∗d∗x∗log(n)), where *t* is the number of the trees, *d* is the depth of the tree, *x* is the number of non-missing entries in the training data, and *n* is the number of training samples. For model inference, XGBoost operates in O(t∗d) in the worst case and O(t∗log(l)) for a balanced tree, where *l* is number of leaves in a tree. The algorithm employed a histogram-based split method to optimize its computational cost. XGBoost also introduces a sparsity-aware approach for parallel tree learning [39]. This characteristic makes XGBoost a useful candidate for real-time applications such as for IDS deployment in IoT environment.

#### 3.3.4. LightGBM

Light Gradient Boosting Machine (LightGBM) is a high-performance ensemble algorithm for gradient boosting that specializes in speed and efficiency. It constructs decision trees sequentially by optimizing an objective function. A key innovation in LightGBM is the leaf-wise tree growth algorithm, which splits the leaf with the highest loss reduction, as opposed to level-wise splitting. This approach yields deeper trees and better accuracy while maintaining computational efficiency [40]. The worst-case computational complexity of LightGBM is O(t∗n∗b∗d), where *t* is the number of trees, *n* is the number of training samples, *b* is the number of histogram bins, and *d* is the maximum depth of each tree. The worst-case inference time takes O(t∗d) while for a balanced tree, it has O(t∗log(l)), where *l* is the number of leaves per tree. The use of histogram binning and leaf-wise growth makes LightGBM a faster algorithm to train large datasets, making it applicable for real-time applications like IDS for IoT environments [40].

#### 3.3.5. CatBoost

Categorical Boosting (CatBoost) is a gradient boosting framework designed to handle categorical data efficiently without requiring extensive preprocessing like one-hot encoding. It builds decision trees sequentially while minimizing a custom loss function. One of the main features of CatBoost is its Ordered Boosting, which prevents over-fitting by using permutation-based sampling during training. It also implements symmetry constraints in tree structures to accelerate learning and maintain robust generalization. Its ease of use and ability to reduce over-fitting make it one of the preferred ensemble algorithms for gradient boosting [41]. Similarly, CatBoost operates in O(t∗n∗b∗d), where *t* is the number of trees, *n* is the number of training samples, *b* is the number of bins (used for feature quantization), and *d* is the maximum depth of each tree. The inference time also takes O(t∗d) in the worst case. CatBoost uses ordered boosting to reduce over-fitting and speeds up training process; it enhances performance for large datasets [42], making it applicable for an IDS in the IoT.

### 3.4. Classification

This study focuses on developing a classification model that can be deployed to detect intrusion in an IoT network. While a majority of the studies in the literature focused on deploying only binary classification approach for IDS, we evaluate the proposed framework using both binary and multi-class classification tasks. The binary classification entails grouping all the attacks in the two datasets into benign and attack categories. The multi-class classification develops ML models to detect different types of attacks such as DDoS, Bot, Infiltration, PortScan, and so on. It is important to mention that we implement both binary and multi-class classification on the CIC-IDS2017 dataset and binary classification on the CICIoT2023 dataset. The purpose is to evaluate the proposed framework across different scenarios while also maintaining promising performance considering the different classification algorithms.

### 3.5. Evaluation Metrics

This study employed different evaluation metrics to evaluate the performance of the proposed framework. We used evaluation metrics such as accuracy, precision, recall, F1-score, AUC-ROC, and MCC. These metrics are computed based on the confusion matrix that consists of true positive (TP), true negative (TN), false positive (FP), and false negative (FN) as the elements of the matrix. TP is the number of attacks that are correctly predicted as attacks. TN is the number of normal (i.e benign) cases that are correctly predicted as normal. FN is the number of attacks that are mistakenly predicted as normal while FP is the number of normal cases that are wrongly predicted as attacks.

Given the different elements of the confusion matrix, several evaluation metrics can be computed as follows. For instance, accuracy is the percentage of correctly classifies attack and normal cases. This is computed by dividing the number of correct predictions over the data by the total number of predictions as follows:

(4) $$Accuracy=\frac{TP+TN}{TP+TN+FP+FN}\;$$

In the context of intrusion detection, precision evaluates how accurately the model identifies actual intrusions among all flagged events. It is calculated by dividing the number of correctly detected intrusions (TP) by the total number of events flagged as intrusions (both TP and FP). A high precision value indicates that the model minimizes false alarms while accurately detecting genuine intrusions. Precision is calculated as:

(5) $$Precision=\frac{TP}{TP+FP}\;$$

Recall metric measures how effectively the model identifies all actual intrusion attempts. It is calculated by dividing the number of correctly detected intrusions (TP) by the total number of actual intrusions, which includes both TP and missed intrusions (FN). A high recall indicates the model’s ability to minimize undetected intrusions, ensuring most security breaches are flagged. Recall is calculated as:

(6) $$Recall=\frac{TP}{TP+FN}\;$$

F1-score is the ratio of recall and precision. It determines the harmonic mean of precision and recall. F1-score is a metric that balances precision and recall to provide a single measure of a model’s performance. It is particularly useful when both FP (false alarms) and FN (missed intrusions) are critical. It is also useful to measure model performance in the presence of imbalanced data distribution, which is the case of the two datasets that we employed in this study. This metric is calculated as:

(7) $$F1-Score=2*\frac{Precision*Recall}{Precision+Recall}\;$$

The AUC-ROC metric is the area under the ROC curve. It sums up how well a model can produce relative scores to discriminate between intrusion or normal events across all classification thresholds. The value of AUC-ROC ranges between 0 and 1, where 0.5 means a random guess, and 1 signifies a perfect prediction result. The last performance metric, MCC, is a statistical metric that evaluates the quality of classification models by considering all elements of the confusion matrix (i.e., TP, TN, FP, and FN). It provides a balanced measure even for imbalanced datasets, as it accounts for the proportionality of intrusion and normal events. The MCC yields a value between −1 and +1, where +1 indicates perfect predictions, 0 suggests no better accuracy than random guessing, and −1 reflects total disagreement between predictions and actual outcomes. This metric is calculated as:

(8) $$MCC=\frac{TP*TN-FP*FN}{\sqrt{\left(TP+FP)*(TP+FN)*(TN+FP)*(TN+FN\right)}}$$

In addition, we reported the False-Positive Rate (FPR) and False-Negative Rate (FNR) of each model. We also computed the training and inference time to evaluate the applicability of the models for IoT environments.

### 3.6. Model Explanation

This study integrates model explainability in the proposed framework. It extends existing studies by providing model transparency and explainability, a feature that has been less addressed by the existing studies on IDS in IoT. To achieve this goal, we integrate rule induction for model explanation in the proposed framework (see Figure 1. Explainable Artificial Intelligence (XAI) is the field of AI that provides explanations for model decisions [43]. This is to enforce transparency and trust as well as to promote human-understandable explanations for decision support. This study adopts a rule-induction method to provide model explanation. Rule induction is a ML technique used to extract interpretable and human-readable rules from datasets or ML models [44,45]. These rules, often in the form of **if–then** statements, describe patterns and relationships within the data. The goal of rule induction is to create a concise set of rules that accurately represent the decision-making process while maintaining clarity.

By transforming data or model outcomes into understandable insights, this will promote transparency and trust regarding what constitute intrusion or normal events. Since the ensemble algorithms considered in this study employed decision trees as their weak learners, we integrate Algorithm 1 into the proposed framework, which induces rules from a decision-tree model. This helps us to gain better understanding of the decisions from the classification algorithms in the form of **if–then** rules.
**Algorithm 1:** Rule Induction for Model Explanation
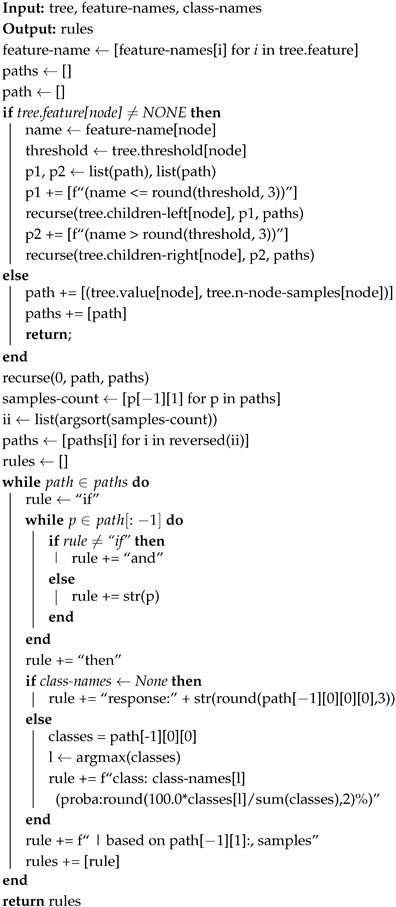


Algorithm 1 begins by taking the trained decision-tree model (i.e., tree), features used to train the model, and the target classes. In the case of binary classification, the target class is either 0 or 1 (i.e., benign or attack). A list containing all the feature names extracted from the trained decision-tree model is created along with two variables to keep track of the rules. A recursive process is then established to traverse both the left and right parts of the decision tree. The process continues until all the features have been expanded. The generated paths in the form of list are iterated to produce concatenated **if–then** rules with their corresponding number of samples covered by the rules, as well as the accuracy of coverage in the form of probabilities. In terms of computational complexity, Algorithm 1 operates in O(n∗d), where *n* is the number of nodes and *d* is the depth of the tree. Since we leverage the built-in feature selection of the decision-tree algorithm, it is expected that the algorithm will use minimal number of features to construct the decision tree for rule induction, which will speed up the rule-extraction process. In addition, the maximum depth of the tree was controlled during the modeling stage based on the hyperparameter of the ensemble algorithm to reduce model complexity. Hence, the rule-induction algorithm is applicable in IoT environments. This approach enables us to provide explanations for the decision to classify an event as intrusion or benign from the trained decision-tree model. We validate the algorithm in Section 4.2.3.

### 3.7. Experimental Setup

In this study, different experiments were conducted to ascertain the efficacy of the proposed framework. Regarding the hyperparameters of each classification algorithm, we set the number of estimators of the ensemble algorithms to 100. The learning rate of AdaBoost was set to 1. XGBoost max-depth, subsample, and eta were set to 6, 0.5, and 0.3, respectively. The remaining hyperparameters of the algorithms were used in their default settings. These configurations yielded promising results in our case. The data were split into a 70/30 train–test ratio, where 70% was used to train the ensemble algorithms and 30% was used for testing.

As stated earlier, this study evaluated the performance of the proposed framework based on two publicly available IoT datasets for intrusion detection. All experiments have been performed on MacBook Pro with Apple M2 chip and 16 GB RAM. The ML algorithms were implemented in Conda environment.

## 4. Results and Discussion

This section presents the results of the different experiments conducted to ascertain the efficacy of the proposed framework. We first present the results of the experiments conducted on the two datasets based on binary and multi-class classification. This is followed with the results of the model explainability for both intrusion and normal cases. We also compare the results of our proposed framework with the state-of-the-art methods.

### 4.1. Results Based on Binary and Multi-Class Classification

In this section, we compare the performance of the different ML classifiers when all the features in the datasets have been used to develop the IDS models. The results obtained show promising performance across the different ensemble-classification algorithms and for the different evaluation metrics (see for example Table 3, Table 4, Table 5 and Table 6). For instance, the results of the binary classification on both CIC-IDS2017 and CICIoT2023 datasets in Table 3 and Table 5 confirmed that the XGBoost ensemble algorithm produced the best results, outperforming the other classifiers for developing IDS that can detect benign and attack scenarios in the two datasets. XGBoost produced an accuracy, precision, recall, F1-score, AUC-ROC, and MCC of 99.91%, 99.91%, 99.91%, 99.91%, 99.88%, and 99.69%, respectively. A similar result was also obtained for the CICIoT2023 dataset where XGBoost produced an accuracy, precision, recall, F1-score, AUC-ROC, and MCC of 98.54%, 98.56%, 98.54%, 98.55%, 93.06%, and 84.83%, respectively. The results of XGBoost, LightGBM and CatBoost are very close for the CIC-IDS2017 dataset based on the binary classification. In the case of CICIoT2023, XGBoost and Random Forest produced competing results across the different evaluation metrics. These results underscore the benefits of using ensemble models for IDS development when the patterns of anomalies are already established in the training datasets.

The ensemble models show interesting results when training and inference time were computed. Using CIC-IDS2017, the results show that LightGBM has the least training time, followed by CatBoost and XGBoost, while XGBoost possesses the least inference time of 0.31 s, indicating that the model is an ideal candidate for deployment in IoT settings (see Table 4). Random Forest has the highest training time among the models. In addition, using CIC-IDS2017, Random Forest, CatBoost, and XGBoost have reduced FPR. However, XGBoost produced the least FNR according to Table 4. Using CICIoT2023, we recorded slightly higher FPR across the models, with XGBoost producing the least FRP, FNR, and model inference time according to Table 6. The implication of this result can be attributed to the distribution of normal cases compared to attack cases in the CICIoT2023 dataset. Nevertheless, XGBoost produced the least inference time and false alarms when compared with the other models. The FPR and FNR are calculated by the averaging method based on the different classes.

For the binary classification task to detect benign or attack cases, despite the fact that the worst-performing classification algorithm is AdaBoost, this classifier still produced competing results with the other ensemble classifiers, showing the applicability of the different ensemble models. The boosting classifiers attempted to correct the prediction made by the previous decision tree model in a sequential manner, thus providing an avenue to develop a scenario where an error from the previous learner is corrected by the next learner until the model finally produces better results.

In a similar manner, the results of the ensemble classifiers for the multi-class classification, as demonstrated in Table 7, further show that the XGBoost classifier still outperformed the other classification models, producing an accuracy, precision, recall, F1-score, AUC-ROC, and MCC of 99.88%, 99.87%, 99.88%, 99.87%, 99.99%, and 99.61%, respectively, based on CIC-IDS2017 dataset. This result was followed by the result of the Random Forest, which produced an accuracy, precision, recall, F1-score, AUC-ROC, and MCC of 99.83%, 99.82%, 99.83%, 99.83%, 99.09%, and 99.44% respectively. Interestingly, the result of LightGBM for the multi-class classification task is lower than the AdaBoost. This result deviates from the binary classification task for IDS where AdaBoost produced the least results based on the different evaluation metrics. The implication of this result further highlights the necessity for evaluating different algorithms for IDS development in order to ascertain the performance of the classifiers based on the different learning tasks. In our case, for the multi-class classification on CIC-IDS2017, XGBoost, Random Forest, and CatBoost show promising performance, with the highest results obtained in the case of XGBoost (see Table 7). The results in Table 8 show that LightGBM has the least training time; however, XGBoost produced the least inference time (3.18 s). When considering model deployment in real time and according to Table 8, XGBoost can be seen as the ideal candidate, since it produces the least inference time and error rates among the ensemble models. Therefore, in the subsequent section, we discuss the results of model explainability based on XGBoost model.

### 4.2. Results Based on Model Explainability

As discussed in the previous section, this study integrates a rule-induction method for model explainability based on the procedures in Algorithm 1. The algorithm extracts decisions from the classifier used by the XGBoost model (i.e, decision tree (DT)). According to Algorithm 1, the rules induced covered the samples for each class category, as well as the probability measures that evaluate the accuracy of the rules. We carried out a model explanation based on binary and multi-class cases. More clearly, the class indicated in each rule specifies a numeric value assigned to the class (e.g., 0 for benign and 1 for attack in the case of binary classification). The subsequent sections further discuss the explainability parts in relation to both binary and multi-class intrusion detection scenarios.

#### 4.2.1. Rule Induction Based on Binary Classification

This section discusses the rules induced based on binary classification task for intrusion detection. Table 9 shows the features that were selected by the algorithm to explain the decision of the classifier behind the binary classification and Table 10 presents the 10 selected rules extracted based on Algorithm 1 using CIC-IDS2017. A total of 39 rules were extracted in the case of binary classification using CIC-IDS2017. In that case, 21 features have been internally selected by the algorithm, as shown in Table 9. For CIC-IoT2023, 32 rules were induced with 11 features internally selected by the algorithm according to Table 9. Thus, we rely on the built-in feature-selection algorithm. We experimented with some external feature-selection methods, but the gain was not significant. The features are briefly described below. The features in the CIC-IDS2017 were extracted using CICFlowMeter, which is capable of extracting features relating to both forward and backward direction of network traffic-flow data [30]. CICFlowMeter can extract the statistical time-related features in these two directions.

- ActiveStd: The standard deviation time a flow was active before becoming idle.
- AveragePacketSize: The average size of the data packet.
- BwdIATMean: The mean time between two packets sent in the backward direction.
- BwdIATStd: The standard deviation time between two packets sent in the backward direction.
- BwdPacketLengthMean: The mean size of packet in backward direction.
- BwdPacketLengthStd: The standard deviation size of a packet in the backward direction.
- DestinationPort: The destination port where the packet was sent.
- FwdIATMax: The maximum time between two packets sent in the forward direction.
- FwdPacketLengthMean: The mean size of a packet in the forward direction.
- FwdPacketLengthStd: The standard deviation size of packet in the forward direction.
- IdleMax: The mean time a flow was idle before becoming active.
- IdleMin: The minimum time a flow was idle before becoming active.
- Init_Win_bytes_backward: The total number of bytes sent in initial window in the backward direction.
- Init_Win_bytes_forward: The total number of bytes sent in initial window in the forward direction.
- MaxPacketLength: The maximum length of a packet.
- PSHFlagCount: The number of packets with PUSH.
- PacketLengthStd: The standard deviation length of a packet.
- SubflowBwdPackets: The average number of packets in a sub flow in the backward direction.
- SubflowFwdBytes: The average number of bytes in a sub flow in the forward direction.
- SubflowFwdPackets: The average number of packets in a sub flow in the forward direction.
- TotalFwdPackets: The total packets in the forward direction.
- HTTPS: If the application layer protocol is HTTPS, or not.
- Max: The maximum packet length in the network flow.
- Min: The minimum packet length in the network flow.
- Number: The number of packets in the network flow.
- Rate: The rate of packet transmission in a network flow.
- Time To Live: The time-to-live (ttl) as the duration of time that packet spent in the network.
- Totsize: The length of the packet.
- FIN Flag Number: The FIN flag value from the TCP network connection.
- PSH Flag Number: The push flag value from the TCP network connection.
- SYN count: The number of packets with SYN flag set in the same flow.
- SYN Flag Number: The SYN flag value from the TCP network connection.

It is important to mention that the threshold values used by each rule are based on the normalized values as described in Equation (1). These thresholds are used by the decision tree to split each node of the tree. For example, considering the first rule in Table 10, it can be seen that the features such as BwdPacketLengthStd, AveragePacketSize, DestinationPort, ActiveStd, BwdIATStd, and BwdPacketLengthStd all contributed to detecting benign events with a probability of 99.72% covering about 1,201,474 samples. The values of each threshold was extracted to provide a better explanation on what constitutes the decision of the classifier corresponding to the first rule. Unlike in most of the previous studies that focused only on detecting intrusion without providing an explanation of the different cases, our approach extracted knowledge from the trained classifier to provide an explanation of the decision made by the classifier (i.e., to determine if an event is an intrusion or not). This is particularly useful to promote trust and model transparency.

For the attack scenario, the second rule in Table 10 shows that features such as BwdPacketLengthStd, DestinationPort, Init_Win_bytes_backward, and Init_Win_bytes_forward with their respective threshold values can be used to identify attack cases covering about 169,254 samples with the probability of 100% on CIC-IDS2017 dataset. Similar results can be seen in the case of CICIoT2023 dataset (see Table 11).

#### 4.2.2. Rule Induction Based on Multi-Class Classification

Interestingly, more explanations were captured by different rules for the multi-class classification to detect intrusion on CIC-IDS2017 dataset, leading to a total of 41 rules. For this specific case of detecting different attack categories, 28 features were internally selected by the DT algorithm. These features are ActiveMax, ActiveStd, AveragePacketSize, BwdIATMean, BwdIATStd, BwdPacketLengthMax, BwdPacketLengthMean, BwdPacketLengthStd, BwdPackets, DestinationPort, FlowIATMax, FlowIATMean, FlowIATMin, FlowIATStd, FwdIATStd, FwdPacketLengthMax, FwdPacketLengthMean, FwdPacketLengthStd, IdleMean, Init_Win_bytes_forward, PSHFlagCount, PacketLengthMean, PacketLengthStd, SubflowBwdBytes, SubflowFwdPackets, TotalBackwardPackets, TotalLengthofBwdPackets, and TotalLengthofFwdPackets.

These rules explain the decision boundary for each category of event, unlike the binary classification that considered both benign and attack cases only. For instance, according to Table 12, rule number two reveals that features such as BwdPacketLengthStd, FwdPacketLengthMax, AveragePacketSize, FwdIATStd, and TotalBackwardPackets with their respective threshold values can be used to detect DoS Hulk attack with 100% probability, covering about 98,318 samples in the training data. Similarly, the third rule shows that leveraging features like BwdPacketLengthStd, AveragePacketSize, PacketLengthStd, BwdPackets, PSHFlagCount, and SubflowFwdPackets, we can detect malicious PortScan attack with 98.94% probability, covering about 63,212 samples. Providing a model explanation such as those presented in this study can guide IoT security analysts and end-users to make an informed decision when detecting intrusion on the networks. This ensures there is transparency in the model decision and also promotes trustworthiness concerning the decision of the IDS system to flag an event as normal or intrusion.

#### 4.2.3. Rule Validation

In this section, we evaluate the performance of the induced rules using the test sets. The test sets are the data that were not used during the model training. This enables us to test how the proposed framework will perform for real-world IDS deployment in IoT. We computed the precision for the rules on the test sets to further show the applicability and effectiveness of the proposed approach in a real-life situation (i.e., using unseen samples for rule validation). The purpose is to further assess the usefulness of the rules based on unseen data. This is particularly crucial to evaluate the applicability of the explainable component of the proposed framework for real-time IDS deployment. Therefore, we validate the rules listed in Table 10, Table 11 and Table 12. Recall that these are selected rules from the total induced rules by the proposed framework as discussed in Section 4.2.

As shown in Table 13, the rule precision produced promising results in the test sets, which further confirmed the trustworthiness and applicability of the rule-induction method for model explanation. The precision of the rules was computed according to Equation (5). The rule validation is essential to demonstrate that the rule-induction method is effective in identifying patterns or anomalies in a practical context. For example, measuring precision shows how often a rule correctly identifies true positives (e.g., actual security threats) out of all instances it flags, providing a clear indicator of its usefulness not only to security analysts and other stakeholders, but also to end-users of smart homes.

### 4.3. Comparison with Existing Studies

In this section, we compare our approach with related studies to provide a discussion on the performance of the proposed framework. Since our proposal extends the body of knowledge by providing an explainable component in the proposed framework; therefore, only the IDS results will be compared to provide some level of fairness with existing studies during comparison. Thus, we compare our results with four studies that used the same datasets as in our case. These studies include [3,13,31,46]. The performance of each model in comparison with our proposed approach (i.e., based on XGBoost) is shown in Table 14. It is important to mention the experimental settings used in each study for clarification on the different results. For instance, the study in [3] used the same train–test splitting ratio as in our case, which is based on 70–30%, respectively. Meanwhile, the studies in [31,46] used an 80–20% ratio for the splitting and the authors did not report whether the over 20 million duplicate samples in the CICIoT2023 dataset were removed or not during their experiments (see, for instance, the study in [31]). However, in our case, we removed all the 20 million duplicate samples, which is particularly beneficial to prevent bias and model over-fitting during the experiments [35,36]. The study in [3] removed 2867 samples that contained NAN and infinity, whereas, in our case, we filled all the missing values in the two datasets by applying kNN imputation with two neighbours. As shown in Table 14, our approach outperformed the study in [3] and equally achieved a comparable performance in terms of accuracy with the study in [31,46]. Furthermore, in terms of precision and F1-score, the proposed approach outperformed the study in [31,46].

### 4.4. Limitations of the Study

Although this study introduces an IDS framework that integrates ensemble learning with rule-induction methods for enhanced model explainability, it is, however, important to mention some limitations of the study. The scalability of our approach to large IoT networks and its performance in massive deployments remain untested. Additionally, the performance metrics, like inference time, might vary across different IoT deployments with heterogeneous resource-constrained devices.

## 5. Conclusions and Future Work

This paper addressed the challenges of detecting intrusion within IoT network by proposing a framework that incorporates ensemble learning and model explainability to address both binary and multi-class classification problems. This study contributes a scalable, explainable IDS framework for IoT networks. Five ensemble algorithms, namely, Random Forest, AdaBoost, XGBoost, LightGBM, and CatBoost, have been studied. To address model explainability, this study integrates a rule-induction method into the proposed framework. The model explainability is based on rule induction from a decision tree, which is the core weak learner of the ensemble algorithms that were considered in this study. Through rigorous experiments to ascertain the performance of the proposed framework, the results show that XGBoost classifier produced the best results. The classification results for both binary and multi-class classification on two publicly available intrusion detection datasets further confirmed that the proposed framework maintained good performance based on the different evaluation metrics and as compared with the state-of-the-art studies. The models’ low inference times make the proposed framework suitable for resource-constrained devices.

The induced rules from the rule-induction algorithm will help not only security analysts and other stakeholders in smart homes, but also help the end-users to understand the decisions made by an IDS system under different circumstances, and to identify network intrusions. Model explainability is crucial for providing an understanding of how an IDS system works, and of how its decisions are made. Our method promotes a better accuracy and a higher level of transparency in the presence of different attack scenarios (i.e., for both binary and multi-class cases).

However, the scalability of our approach to massive IoT networks remains untested. Future work should focus on deep learning methods in connection to Local Interpretable Model-agnostic Explanations (LIME) and SHapley Additive exPlanations (SHAP). The possibility of integrating LIME and SHAP with our proposed framework can be investigated in future research, since both methods provide model-agnostic explanations. In addition, we will focus on exploring more IoT datasets to broaden the understanding of the different attacks and how to effectively explain their patterns based on a robust model explainability approach. Furthermore, a more user-friendly way of presenting attack explanations, such as natural language descriptions, could be interesting to consider.

## Figures and Tables

**Table 1:** Class distribution in the CIC-IDS2017 dataset.

Label	Count	Percentage (%)
BENIGN	2,273,097	0.803004
DoS Hulk	231,073	0.081630
PortScan	158,930	0.056144
DDoS	128,027	0.045227
DoS GoldenEye	10,293	0.003636
FTP-Patator	7938	0.002804
SSH-Patator	5897	0.002083
DoS slowloris	5796	0.002048
DoS Slowhttptest	5499	0.001943
Bot	1966	0.000695
Web Attack Brute Force	1507	0.000532
Web Attack XSS	652	0.000230
Infiltration	36	0.000013
Web Attack Sql Injection	21	0.000007
Heartbleed	11	0.000004

**Table 2:** Class distribution in the CICIoT2023 dataset.

Label	Count	Percentage (%)
DDOS-ICMP-FLOOD	6,893,259	15.312
DDOS-UDP-FLOOD	5,181,027	11.508
DDOS-TCP-FLOOD	4,306,086	9.565
DDOS-PSHACK-FLOOD	3,920,372	8.708
DDOS-SYN-FLOOD	3,886,130	8.632
DDOS-RSTFINFLOOD	3,872,808	8.603
DDOS-SYNONYMOUSIP_FLOOD	3,445,659	7.654
DOS-UDP-FLOOD	3,177,323	7.058
DOS-TCP-FLOOD	2,558,256	5.683
DOS-SYN-FLOOD	1,942,176	4.314
BENIGN	1,051,373	2.335
MIRAI-GREETH-FLOOD	949,381	2.109
MIRAI-UDPPLAIN	852,695	1.894
MIRAI-GREIP-FLOOD	719,655	1.599
DDOS-ICMP-FRAGMENTATION	433,157	0.962
VULNERABILITYSCAN	357,583	0.794
MITM-ARPSPOOFING	294,469	0.654
DDOS-UDP-FRAGMENTATION	274,909	0.611
DDOS-ACK-FRAGMENTATION	272,793	0.606
DNS-SPOOFING	171,468	0.381
RECON-HOSTDISCOVERY	128,677	0.286
RECON-OSSCAN	93,970	0.209
RECON-PORTSCAN	78,730	0.175
DOS-HTTP-FLOOD	68,799	0.153
DDOS-HTTP-FLOOD	27,597	0.061
DDOS-SLOWLORIS	22,400	0.050
DICTIONARYBRUTEFORCE	12,522	0.028
BROWSERHIJACKING	5630	0.013
COMMANDINJECTION	5168	0.011
SQLINJECTION	5022	0.011
XSS	3705	0.008
BACKDOOR-MALWARE	3078	0.007
RECON-PINGSWEEP	2161	0.005
UPLOADING-ATTACK	1196	0.003

**Table 3:** Results based on binary classification with all feature sets using CIC-IDS2017 dataset.

Algorithm	Accuracy	Precision	Recall	F1-Score	AUC-ROC	MCC
Random Forest	0.9987	0.9987	0.9986	0.9987	0.9973	0.9953
AdaBoost	0.9938	0.9938	0.9938	0.9938	0.9871	0.9779
**XGBoost**	**0.9991**	**0.9991**	**0.9991**	**0.9991**	**0.9988**	**0.9969**
LightGBM	0.9990	0.9990	0.9990	0.9990	0.9986	0.9966
CatBoost	0.9990	0.9990	0.9990	0.9990	0.9986	0.9966

**Table 4:** Results of binary classification with all feature sets using CIC-IDS2017 dataset based on training time, inferencing time, and error rates.

Algorithm	Training Time (s)	Inference Time (s)	FPR	FNR
Random Forest	338.24	7.66	0.00060	0.00488
AdaBoost	244.76	8.27	0.00271	0.02308
XGBoost	105.84	0.31	0.00073	0.00162
LightGBM	5.46	0.84	0.00074	0.00198
CatBoost	24.20	13.71	0.00072	0.00207

**Table 5:** Results based on binary classification with all feature sets using CICIoT2023 dataset.

Algorithm	Accuracy	Precision	Recall	F1-Score	AUC-ROC	MCC
Random Forest	0.9852	0.9854	0.9853	0.9854	0.9264	0.8459
AdaBoost	0.9802	0.9808	0.9802	0.9804	0.9118	0.7964
**XGBoost**	**0.9854**	**0.9856**	**0.9854**	**0.9855**	**0.9306**	**0.8483**
LightGBM	0.9846	0.9849	0.9846	0.9848	0.9275	0.8407
CatBoost	0.9843	0.9844	0.9843	0.9843	0.9227	0.8358

**Table 6:** Results of binary classification with all feature sets using CICIoT2023 dataset based on training time, inferencing time, and error rates.

Algorithm	Training Time (s)	Inference Time (s)	FPR	FNR
Random Forest	618.02	19.32	0.14082	0.00810
AdaBoost	922.97	28.36	0.17134	0.01191
XGBoost	205.74	0.54	0.13024	0.00850
LightGBM	8.84	1.04	0.13683	0.00894
CatBoost	70.25	18.88	0.14836	0.00917

**Table 7:** Results based on multi-class classification with all feature sets using CIC-IDS2017 dataset.

Algorithm	Accuracy	Precision	Recall	F1-Score	AUC-ROC	MCC
Random Forest	0.9983	0.9982	0.9983	0.9983	0.9974	0.9944
AdaBoost	0.8911	0.8072	0.8911	0.8462	0.6362	0.5837
**XGBoost**	**0.9988**	**0.9987**	**0.9988**	**0.9987**	**0.9999**	**0.9961**
LightGBM	0.8100	0.7947	0.8100	0.7966	0.5517	0.3269
CatBoost	0.9984	0.9983	0.9984	0.9982	0.9861	0.9946

**Table 8:** Results of multi-class classification with all feature sets using CIC-IDS2017 dataset based on training time, inferencing time, and error rates.

Algorithm	Training Time (s)	Inference Time (s)	FPR	FNR
Random Forest	376.57	12.11	0.00038	0.19146
AdaBoost	262.82	15.04	0.03136	0.87400
XGBoost	1055.55	3.18	0.00018	0.14350
LightGBM	30.98	5.67	0.03727	0.87911
CatBoost	235.89	14.31	0.00028	0.20642

**Table 9:** Selected features by the DT algorithm based on binary classification.

CIC-IDS2017	CIC-IoT2023
ActiveStd	HTTPS
AveragePacketSize	Max
BwdIATMean	Min
BwdIATStd	Number
BwdPacketLengthMean	Rate
BwdPacketLengthStd	Time_To_Live
DestinationPort	Totsize
FwdIATMax	fin_flag_number
FwdPacketLengthMean	psh_flag_number
FwdPacketLengthStd	syn_count
IdleMax	syn_flag_number
IdleMin	
Init_Win_bytes_backward	
Init_Win_bytes_forward	
MaxPacketLength	
PSHFlagCount	
PacketLengthStd	
SubflowBwdPackets	
SubflowFwdBytes	
SubflowFwdPackets	
TotalFwdPackets	

**Table 10:** Ten selected rules induced from binary classification using CIC-IDS2017 dataset.

Rule	RuleID	Class	Probability (%)
if (BwdPacketLengthStd ≤ 0.183) and (AveragePacketSize > 0.002) and (DestinationPort > 0.0) and (ActiveStd ≤ 0.061) and (BwdIATStd ≤ 0.847) and (BwdPacketLengthStd ≤ 0.159) then class: 0 (proba: 99.72%) | based on 1,201,474 samples	1	Benign	99.72
if (BwdPacketLengthStd > 0.183) and (DestinationPort ≤ 0.004) and (Init_Win_bytes_backward ≤ 0.004) and (Init_Win_bytes_backward > 0.003) and (Init_Win_bytes_forward ≤ 0.723) then class: 1 (proba: 100.0%) | based on 169,254 samples	2	Attack	100
if (BwdPacketLengthStd ≤ 0.183) and (AveragePacketSize ≤ 0.002) and (MaxPacketLength ≤ 0.0) and (SubflowFwdPackets ≤ 0.0) and (IdleMin ≤ 0.088) and (DestinationPort > 0.001) then class: 0 (proba: 99.92%) | based on 148,358 samples	3	Benign	99.92
if (BwdPacketLengthStd ≤ 0.183) and (AveragePacketSize ≤ 0.002) and (MaxPacketLength > 0.0) and (SubflowBwdPackets ≤ 0.0) and (Init_Win_bytes_backward ≤ 0.004) and (FwdPacketLengthStd ≤ 0.0) then class: 1 (proba: 96.01%) | based on 103,209 samples	4	Attack	96.01
if (BwdPacketLengthStd ≤ 0.183) and (AveragePacketSize ≤ 0.002) and (MaxPacketLength > 0.0) and (SubflowBwdPackets > 0.0) and (Init_Win_bytes_forward > 0.0) and (FwdIATMax ≤ 0.419) then class: 0 (proba: 98.87%) | based on 44,898 samples	5	Benign	98.87
if (BwdPacketLengthStd ≤ 0.183) and (AveragePacketSize ≤ 0.002) and (MaxPacketLength > 0.0) and (SubflowBwdPackets ≤ 0.0) and (Init_Win_bytes_backward > 0.004) and (PacketLengthStd > 0.0) then class: 0 (proba: 100.0%) | based on 16,821 samples	6	Benign	100
if (BwdPacketLengthStd ≤ 0.183) and (AveragePacketSize ≤ 0.002) and (MaxPacketLength ≤ 0.0) and (SubflowFwdPackets > 0.0) and (TotalFwdPackets ≤ 0.0) and (DestinationPort ≤ 0.004) then class: 1 (proba: 91.93%) | based on 5540 samples	7	Attack	91.93
if (BwdPacketLengthStd ≤ 0.183) and (AveragePacketSize > 0.002) and (DestinationPort ≤ 0.0) and (BwdIATMean ≤ 0.002) and (Init_Win_bytes_forward > 0.004) and (Init_Win_bytes_backward ≤ 0.004) then class: 0 (proba: 100.0%) | based on 6203 samples	8	Benign	100
if (BwdPacketLengthStd ≤ 0.183) and (AveragePacketSize ≤ 0.002) and (MaxPacketLength > 0.0) and (SubflowBwdPackets > 0.0) and (Init_Win_bytes_forward ≤ 0.0) and (DestinationPort ≤ 0.004) then class: 1 (proba: 99.93%) | based on 2896 samples	9	Attack	99.93
if (BwdPacketLengthStd ≤ 0.183) and (AveragePacketSize ≤ 0.002) and (MaxPacketLength > 0.0) and (SubflowBwdPackets > 0.0) and (Init_Win_bytes_forward ≤ 0.0) and (DestinationPort > 0.004) then class: 0 (proba: 100.0%) | based on 1789 samples	10	Benign	100

**Table 11:** Ten selected rules induced from binary classification using CICIoT2023 dataset.

Rule	RuleID	Class	Probability (%)
if (Number ≤ 0.096) and (Max > 0.028) and (psh_flag_number ≤ 0.317) and (HTTPS > 0.317) and (Time_To_Live > 0.248) and (syn_flag_number ≤ 0.05) then class: 0 (proba: 90.73%) | based on 164,910 samples	11	Benign	90.73
if (Number > 0.096) then class: 1 (proba: 100.0%) | based on 7,529,780 samples	12	Attack	100
if (Number ≤ 0.096) and (Max ≤ 0.028) and (HTTPS > 0.268) and (Max > 0.028) and (Max ≤ 0.028) and (Totsize > 0.019) then class: 1 (proba: 100.0%) | based on 42,843 samples	13	Attack	100
if (Number ≤ 0.096) and (Max ≤ 0.028) and (HTTPS ≤ 0.268) and (HTTPS ≤ 0.05) and (syn_flag_number > 0.183) and (Totsize ≤ 0.004) then class: 1 (proba: 99.85%) | based on 33,640 samples	14	Attack	99.85
if (Number ≤ 0.096) and (Max ≤ 0.028) and (HTTPS ≤ 0.268) and (HTTPS ≤ 0.05) and (syn_flag_number ≤ 0.183) and (Min ≤ 0.001) then class: 1 (proba: 100.0%) | based on 16,885 samples	15	Attack	100
if (Number ≤ 0.096) and (Max > 0.028) and (psh_flag_number ≤ 0.317) and (HTTPS > 0.317) and (Time_To_Live ≤ 0.248) and (HTTPS ≤ 0.95) then class: 0 (proba: 86.02%) | based on 15,937 samples	16	Benign	86.02
if (Number ≤ 0.096) and (Max ≤ 0.028) and (HTTPS > 0.268) and (Max ≤ 0.028) and (Time_To_Live ≤ 0.619) and (Min ≤ 0.001) then class: 1 (proba: 100.0%) | based on 14,997 samples	17	Attack	100
if (Number ≤ 0.096) and (Max ≤ 0.028) and (HTTPS ≤ 0.268) and (HTTPS > 0.05) and (Min ≤ 0.001) then class: 1 (proba: 100.0%) | based on 14,643 samples	18	Attack	100
if (Number ≤ 0.096) and (Max > 0.028) and (psh_flag_number > 0.317) and (Totsize > 0.096) and (Time_To_Live ≤ 0.235) and (HTTPS > 0.65) then class: 1 (proba: 98.13%) | based on 5357 samples	19	Attack	98.13
if (Number ≤ 0.096) and (Max > 0.028) and (psh_flag_number > 0.317) and (Totsize > 0.096) and (Time_To_Live > 0.235) and (Max > 0.084) then class: 0 (proba: 90.99%) | based on 966 samples	20	Benign	90.99

**Table 12:** Ten selected rules induced from multi-class classification using CIC-IDS2017 dataset.

Rule	RuleID	Class	Probability (%)
if (BwdPacketLengthStd ≤ 0.183) and (AveragePacketSize > 0.002) and (DestinationPort > 0.0) and (ActiveStd ≤ 0.061) and (BwdIATStd ≤ 0.847) then class: 0 (proba: 99.66%) | based on 1,204,361 samples	21	Benign	99.66
if (BwdPacketLengthStd > 0.183) and (FwdPacketLengthMax > 0.002) and (AveragePacketSize > 0.169) and (FwdPacketLengthMax ≤ 0.017) and (FwdIATStd > 0.068) and (TotalBackwardPackets ≤ 0.0) then class: 4 (proba: 100.0%) | based on 98,318 samples	22	DoS Hulk	100
if (BwdPacketLengthStd ≤ 0.183) and (AveragePacketSize ≤ 0.002) and (PacketLengthStd > 0.0) and (BwdPackets > 0.003) and (PSHFlagCount > 0.5) and (SubflowFwdPackets ≤ 0.0) then class: 10 (proba: 98.94%) | based on 63,212 samples	23	PortScan	98.94
if (BwdPacketLengthStd > 0.183) and (FwdPacketLengthMax ≤ 0.002) and (FwdPacketLengthMean > 0.001) then class: 2 (proba: 100.0%) | based on 56,497 samples	24	DDoS	100
if (BwdPacketLengthStd > 0.183) and (FwdPacketLengthMax > 0.002) and (AveragePacketSize > 0.169) and (FwdPacketLengthMax ≤ 0.017) and (FwdIATStd ≤ 0.068) and (FlowIATMax ≤ 0.02) then class: 4 (proba: 99.13%) | based on 9842 samples	25	DoS Hulk	99.13
if (BwdPacketLengthStd ≤ 0.183) and (AveragePacketSize > 0.002) and (DestinationPort > 0.0) and (DestinationPort ≤ 0.0) and (FwdPacketLengthMax ≤ 0.022) and (Init_Win_bytes_forward > 0.004) then class: 0 (proba: 100.0%) | based on 4194 samples	26	Benign	100
if (BwdPacketLengthStd > 0.183) and (FwdPacketLengthMax > 0.002) and (AveragePacketSize > 0.169) and (FwdPacketLengthMax ≤ 0.017) and (FwdIATStd ≤ 0.068) and (FlowIATMax > 0.02) then class: 3 (proba: 97.23%) | based on 2093 samples	27	DoS GoldenEye	97.23
if (BwdPacketLengthStd ≤ 0.183) and (AveragePacketSize > 0.002) and (DestinationPort > 0.0) and (DestinationPort ≤ 0.0) and (FwdPacketLengthMax > 0.022) then class: 11 (proba: 100.0%) | based on 2077 samples	28	SSH-Patator	100
if (BwdPacketLengthStd ≤ 0.183) and (AveragePacketSize > 0.002) and (DestinationPort > 0.0) and (DestinationPort > 0.0) and (ActiveStd > 0.061) and (BwdIATMean > 0.192) then class: 6 (proba: 93.78%) | based on 1286 samples	29	DoS slowloris	93.78
if (BwdPacketLengthStd ≤ 0.183) and (AveragePacketSize > 0.002) and (DestinationPort ≤ 0.0) and (BwdPacketLengthMax ≤ 0.002) and (FwdPacketLengthStd ≤ 0.001) and (FwdPacketLengthMean > 0.002) then class: 7 (proba: 100.0%) | based on 5 samples	30	FTP-Patator	100

**Table 13:** Rule validation using test sets.

RuleIDs	Dataset	Task	Tot. Samples	Normal	Attack	Precision
R1–R10	CIC-IDS2017	Binary	756,709	628,958	127,751	97.57
R11–R20	CICIoT2023	Binary	2,100,574	104,439	1,996,135	99.99
R21–R30	CIC-IDS2017	Multiclass	756,709	628,958	127,751	89.95

**Table 14:** A comparison of the proposed approach with the state-of-the-art studies.

Model	Accuracy	Precision	F1-Score	Dataset	Task
Random Forest [3]	99.67	N/A	N/A	CIC-IDS2017	Binary
XGBoost [**ours**]	99.91	99.91	99.91	CIC-IDS2017	Binary
Random Forest [3]	99.68	N/A	N/A	CIC-IDS2017	Multi-class
XGBoost [**ours**]	99.88	99.87	99.87	CIC-IDS2017	Multi-class
Random Forest [31]	99.68	96.53	96.52	CICIoT2023	Binary
Random Forest [46]	99.68	96.69	96.55	CICIoT2023	Binary
XGBoost [**ours**]	98.54	98.56	98.55	CICIoT2023	Binary

## Data Availability

The two datasets used in this study are publicly available and we have provided the links to download them in the article.
